# New approaches to the neural correlates of consciousness and the unified perception of the mind. Experiments and treatments

**DOI:** 10.3389/fpsyg.2026.1952709

**Published:** 2026-10-06

**Authors:** Tiziano Cantalupi

**Affiliations:** Department of Biosciences, University of Camerino, Camerino, Italy

**Keywords:** biology, biophysics, brain, cells, neuron, neuroscience

## Abstract

This article seeks to answer questions that are currently at the forefront of neuroscientific research. An attempt will be made to assign a numerical value to the *minimum set* of neurons necessary to generate a *mental process* at a given time. Along the same line of enquiry, the number of neurons involved and the time required for the emergence of a mental experience, engaging several areas of the cerebral cortex in parallel, will also be calculated. With the acquired data, we will concurrently explore the *binding problem* and the nature of consciousness. The latter can *also* be understood as the expression of “classical” and non-classical dynamics. The paper concludes by proposing a series of experiments that could be undertaken to help us better understand – and, if necessary, treat – the “neural system” in its entirety.

## Introduction

This article is essentially divided into two distinct parts. In the first part (which comprises Section 1), the subject of analysis will be the determination of the Neural Correlates of Consciousness (NCC). This topic will be approached within the framework of “classical” biophysics and neurobiology. In the second part (which comprises Sections 2 and 3), an attempt will be made to explain various unsolved questions in neuroscience, while also proposing specific experiments to be conducted. These discussions will be set within a context in which “classical” and quantum physics and biology will run in parallel, occasionally intersecting to share findings and insights.

The following three sections form the focus of the research presented in this work:

(1) Identification of the minimum set of neurons, or processed events, necessary to give rise to a specific subjective conscious experience (perception, thought, emotion, etc.).

(2) On the basis of the previous section and drawing on recent experiments in the field of biophysics and quantum biology, a *classical* and *quantum* model of the mind-brain system will be proposed. The average time for a mental process in the NCC calculated in Section 1 will be taken into account. The time and number of neurons involved in a mental experience that “synchronizes” different, separate areas of the brain will also be defined.

(3) The last section will propose a series of experiments to be carried out on live neuron cultures in vitro, which should lead to the identification of the typical resonance frequencies that characterize neuron cells, with potential implications for the treatment of specific brain disorders and other conditions.

## Section 1. Determination of the average value of the neural correlates of consciousness

In this section, an attempt will be made to define an average value for the NCC. The role of synapses will be central to the ideas that will progressively take shape. Indeed, the only way that neurons in a network can “interface” is through synapses. These tiny “buttons” profoundly influence both the processing and transmission of “information” from one neuron to another, also helping to define the minimum number of neurons required to give rise to a subjective conscious experience.

The flow of Sodium ions (which, on closer inspection, is a downstream effect, i.e., after the synapse, of the action potential coming from an “excitatory” neuron), which enters the neurons en masse from the postsynaptic membrane, will be of fundamental importance in defining the value of NCC. It is this flow that essentially coincides with the onset of mental activity.

Thus, the action potential that arrives from the axon of any neuron in a network reaches the presynaptic terminal. Here, a copious flow of calcium ions causes the release of neurotransmitters from the vesicles into the synaptic space. The neurotransmitters then bind to the receptors on the postsynaptic membrane of the next neuron, causing the opening of the ligand-dependent channels which, due to the strong electrochemical gradient, trigger a massive flow of Sodium ions that cross the membrane towards the interior of the receiving neuron. The entry of positive charges (Na^+^) into the neuron depolarises the membrane, making it positive: this phase is known as an excitatory postsynaptic potential (EPSP). From this point onwards, the signals reach the axon hillock, where an *action potential* is generated. This spreads along the axon and, through its terminals, reaches the synapses of the neurons in the network. *Graded potentials* play a fundamental role in this process. Graded potentials coincide with the process of local membrane depolarisation described above (in neurophysiology, this is described as a variation in membrane potential), and although they decrease with distance, they manage to expand within the neuron (adding partially or fully to graded potentials coming from other postsynapses), in an almost “delocalised” form, subsequently reaching the trigger zone of the axon and causing the opening of voltage-dependent channels that trigger the action potential. In our view, graded potentials may be considered the basis of cognitive processes, given that, by spreading throughout the entire soma and interacting with the systems present there, they play a decisive role in the processing and integration of complex information.

Therefore, in this study, we will focus our attention on the entry of Sodium ions into the neurons in the network (over the course of a couple of milliseconds). It is this flow that essentially contributes to the onset of mental activity. Synapses and Na^+^ flow, therefore, together represent the fundamental components for the transfer of “information” between neurons and the means by which, within the neurons themselves, the excitatory nerve impulse that underpins brain processes is generated.

Before dealing directly with the calculations that will allow us to assign a value to the NCC, we must first introduce the topic by presenting the conclusions of a large series of experiments conducted in various parts of the world since 1970 in the fields of biology and neurobiology (Sections 2 and 3 will make frequent reference to these experiments). The first fundamental results of these tests were presented at an important international symposium held in December 1982 in Bad Neuenahr (Fröhlich and Kremer, 1983). Nearly all of the research presented there showed how high-frequency microwave *pulses* (in a range between 42 and 69 GHz) could significantly alter the properties of biological systems in a “non-thermal” way.

It is well-known that electromagnetic radiation acts on living systems through a thermal mechanism, whereby the energy of the incident radiation heats the affected system and this heating alters its properties. In the experiments in question, however, the power levels involved were kept very low to ensure that the total amount of energy supplied was minimal, resulting in negligible heating. Under these conditions, the biological effects observed (morphological changes, protozoan motility, interference with cell growth, etc.) *could only be due to the action of microwaves on the biological system.* The results of these experiments can be summarized as follows: biological effects are observed at specific frequencies of incident radiation. The non-thermal biological effect of radiation does not, however, occur at all possible frequencies, but only at certain frequencies specific to the particular biological material being irradiated. In short, it is a resonance effect.

The selectivity of the radiation frequencies in these experiments strongly echoes the work carried out by prominent biophysicists (Grundler and Keilmann, 1983) notably Fröhlich (1968, 1969, 1970, 1975, 1984, 1986) who, from 1965 to the present day, have suggested that external radiation is only absorbed by the system when it resonates with one of the possible frequencies predicted by the model, activating the biological effects controlled by that specific frequency. Therefore, at the basis of cellular activity, there appears to be a process of “extended coherence” that can be detected through resonance oscillations around 10^10^–10^11^ Hz.

Recent experiments conducted by a group of neuroscientists and physicists from the Universities of Salzburg and Vienna (Bernroider and Roy, 2005; Summhammer et al., 2012, 2018; Bernroider and Summhammer, 2012; Salari et al., 2015), have shown that ions (remaining within the field of neuroscience) “delocalise” as they pass through ion channels, i.e., they extend into space: more like a *coherent wave* than an atom. This ionic wave is thought to oscillate at extremely high frequencies (up to 900 billion Hz) – bearing in mind that classic brain waves such as gamma, beta, alpha, theta and delta brain waves oscillate at a maximum of 200 Hz – all of which would suggest that the ions crossing the neuron membrane are actual waves (with a considerable energy content, moreover) with all the characteristics of waves. The action potential that arises in the axon hillock and reaches the synapses can also be considered, not as a discrete entity, but as “a wave of charges,” or “an electrical wave.”

For the research that forms the basis of the current section, part of Section 2 and all of Section 3 of our article, we will adopt an average oscillation frequency of 50 GHz (5 × 10^10^ Hz) for Na and K ions (whose dynamics underlie much of neuronal activity as a whole).

We will now present a series of elements that will enable us to arrive at the final calculation of NCC. The first of these elements concerns the number of synapses distributed across the entire membrane of an average cortical neuron: according to official estimates, these range from 1,000 to 10,000.

But How many synapses are significant for the purposes of our research?

It is now an established fact that the electrical activity associated with mental processes simultaneously affects all the neurons within a specific area of the brain. The information that travels almost in real time from one neuron to another in a given area (or network) via synapses means that the latter can effectively be considered a single *coherent entity*. Of course, for various reasons, only a certain number of synapses are activated “in phase” (recalling behaviors similar to the dynamics of *coherent waves*). A realistic estimate in our view of the number of “synchronized” synapses per neuron is around 5,000.

The second element to be defined before starting the calculations of NCC is the average number of (Na^+^) selectively “activable” ion channels in the area of a single postsynaptic membrane: we estimate that there are 50. The last element concerns the number of Na^+^ that pass through a single ion channel in a postsynaptic membrane in the space of 1 or 2 ms during the depolarisation phase: we estimate this number to be approximately 8,400. Therefore, the total number of (Na^+^) selectively “activable” ion channels in the postsynaptic membrane areas will be 250,000, which multiplied by 8,400 will give 2.1 × 10^9^, i.e., the total number of Sodium ions entering a neuron during the depolarisation phase, which we will denote with N_tot_.

It should be noted that the choice of the number of synapses, the number of selectively “activable” ion channels in the area of a single postsynaptic membrane, and the definition of the flow of Na^+^ through the membrane was dictated not only by official estimates, but also by a range of other factors that we will explore in more detail in the next section.

Another essential element for calculating the minimum number of neurons necessary for a mental process is what we have called, in perhaps somewhat abstract language, the “coefficient N√N.” This coefficient (explicitly introduced in this work) will allow us to define the diffusion gradient (in parallel) of “nerve impulses” in a specific area of the cortex starting from the value, N_tot_, characterizing the total flow of Sodium ions that, during the depolarisation phase, passes through the areas of the postsynaptic membranes of a single cortical neuron. With these final pieces of information, we have all the elements we need to calculate the NCC.

The first value we will determine is the energy (E) of a single Na^+^, according to the formula E = *h f* (where *h* is Planck’s constant, *f* is the oscillation frequency, which we have estimated to be 5 × 10^10^ Hz), obtaining a value of 3.31 * 10^–23^. The determination of the energy values necessary to achieve the goal of defining the NCC, together with the introduction of the “coefficient N√N,” will be a distinctive feature of this work.

We can now multiply the energy of a single Na^+^ by N_tot_ to obtain the total energy (E_tot_) of the Sodium ions entering a neuron from the postsynapses during the depolarisation phase. This value is approximately 6.95 × 10^–14^.

At this point, the “coefficient N√N” comes into play (where N corresponds to N_tot_) and, as previously indicated, this will allow us to calculate the diffusion gradient of “nerve impulses” (in parallel) in a given cortical area. Therefore N√N (with N = N_tot_) will be approximately 9.62 × 10^13^. Multiplying this number by the energy of a single Na^+^ gives us 3.18 × 10^–9^; this figure represents the “critical” energy of a certain number of neurons involved in a mental process. We can now divide this value by the total energy (E_tot_) of the transient ions in a single neuron. This last calculation leads us directly to the NCC, which in our case corresponds to approximately 45,800 neurons.

## Section 2. Classical and quantum model of the mind. The binding problem and the nature of consciousness

In Section 1, equations derived from “classical” biophysics were essentially used (albeit with a slight adjustment when adopting the value of the oscillation frequency of Sodium ions) to determine the average value of NCC.

In this second section, we will partly abandon “classical” physics and biology to explore mind– brain dynamics and the parameters of NCC, drawing primarily on quantum physics and biology. To this end, we will refer primarily to the work carried out by the group of neuroscientists and physicists at the Universities of Salzburg and Vienna (see Section 1) and the interpretation of quantum mechanics given by the physicists Ghirardi et al. (1986): hereafter referred to as GRW.

Furthermore, numerous studies in the field of quantum biology, ranging from theoretical to experimental, conducted by a large group of researchers in various parts of the world, have demonstrated how superposition, coherence and the quantum tunneling effect (Yuan et al., 2025) are commonly used by living systems, such as birds, plants, enzymes and even proteins.

Why use quantum physics and quantum biology to describe brain dynamics?

Our mental states are, at a certain level, quantum states. Indeed, our brain functions through the propagation of electrical signals within and between neurons; and electrical signals (both in corpuscular and wave form) are ultimately quantum phenomena. The ultramicroscopic size of the ions that characterize neuronal activity undeniably falls within the sphere of influence of quantum mechanics. Moreover, certain aspects of mental dynamics, due to their “non-deterministic” and noncomputational content, seem to be better described by quantum physics, which is intrinsically probabilistic, than by “classical physics,” which is notoriously deterministic and mechanistic.

While the human body is undoubtedly a mechanistic system, the mind – and especially consciousness, due to its specific characteristics – cannot be entirely bound by strict mechanistic principles or the constraints of the principle of cause and effect, but should also be free to manifest itself as a *field of possibilities*.

There are essentially three reasons why certain quantum phenomena should not be able to occur in biological systems at room temperature, particularly within the brain, which is a warm and moist system.

The first reason concerns the size and temperature of the neurons that are responsible for brain activity as a whole. Neurons are of such a size (billions of atoms) and temperature that quantum effects should gradually “fade out.” In liquid helium and cuprates, macroscopic quantum coherence phenomena such as superfluidity and superconductivity have been observed at temperatures close to absolute zero (−270 °C). Macroscopic quantum phenomena involving superconductivity have recently been observed in special ceramics even at “high” temperatures: i.e., −46 °C. In any case, the number of constituents in a set and the temperature seem to strongly influence quantum dynamics.

The second reason that would prevent macroscopic quantum effects in inanimate matter and biological systems is related to the phenomenon known as decoherence (Zurek, 1991, 2003). Systems such as neurons, and by extension the brain, for example, are not isolated systems. They constantly interact with “the environment.” Continuous interaction with the external environment leads quantum systems to lose their characteristics (superposition, coherence, non-locality) and become “classical.” Therefore, certain phenomena such as superposition and coherence are not expected to persist long enough to influence biological dynamics.

The third reason concerns an aspect that derives from the linearity of the Schrödinger equation. The Schrödinger wave equation (denoted by the symbol Ψ) is the basic formula for most quantum calculations and its linearity raises a number of issues.

These three objections to the limits of applicability of quantum mechanics in biological systems are, however, countered by a whole series of studies that have shown how the quantum tunneling effect, superposition and extended coherence are routinely exploited by plants and animals.

In chlorophyll photosynthesis, for example, an extended form of quantum coherence (Lee et al., 2007) expressed through beats (Engel et al., 2007) is maintained for long enough to allow basic biological processes to take place. In 1989, a group led by Judith Klinman of the University of Berkeley demonstrated (Cha et al., 1989) how the quantum tunneling effect of protons plays a fundamental role in ADH enzymatic reactions. Nigel Scrutton’s team (Masgrau et al., 2004, 2006) at the University of Manchester, also conducted experiments similar to Klinman’s with other types of enzymes, demonstrating the existence of kinetic isotope effects due to the tunnel effect. Similar experiments have also shown that the tunneling effect is linked to the development of (protein) structures such as cross-linked helices (Beratan and Skourtis, 1998).

Last but not least, it is worth mentioning the studies that have shown how certain birds use a sort of biological quantum compass to navigate, which depends on pairs of free radicals in quantum coherent correlation, located in their eyes. The avian compass can interact with the Earth’s magnetic field through a resonance effect that occurs only at certain frequencies – something similar to the resonance phenomena discussed in Section 1. In any case, it is interesting to note how coherence can be maintained for a few milliseconds in a warm and humid environment such as a bird’s eye and not be canceled out by decoherence (Gauger et al., 2011). Several studies in this field have shown that quantum processes such as those described above appear to be used by numerous animals, including cockroaches, whales, dolphins, lobsters, manta rays, sharks, bees, etc.

The theoretical and experimental studies just described, together with the work carried out at the Universities of Salzburg and Vienna (mentioned above), which demonstrate in the field of neurobiology that ions in neurons are “delocalised” entities – more waves than “corpuscles” and therefore capable of expressing coherence and quantum superposition – support the thesis that macroscopic quantum phenomena may underlie brain activity and that, even if only for brief moments, these phenomena may escape pervasive decoherence.

Biological systems have probably learned in the course of evolution (more than any other system) to exploit quantum effects such as coherence and non-locality. Scientists such as McFadden and Al-Khalili (1999, 2008), McFadden, 2000, Patel (2001), Kryachko (2002), Godbeer et al. (2015) and many others have amply demonstrated this.

For quantum effects to somehow manifest in systems as complex and warm as living matter, environmental agitation must be able to sustain these effects rather than stifle them. In the presence of thermal agitation, quantum coherence could establish a new mesoscopic scale. Thus, the dynamics between quantum elements with multiple interactions and the environment could, in some way, end up compensating for the effects of decoherence. Therefore, initially disordered/chaotic biological quantum systems could acquire a quantum-ordered (coherent) behavior one of a kind on a mesoscopic scale without isolation from the environment.

Returning to neurobiology, quantum coherence must therefore be able to cross the synaptic barrier between neurons and extend not only to specific cortical areas, but also to the entire brain. The unity of the mind can only arise if there is some form of quantum coherence that connects/synchronizes spatially distant neural areas (with specific functions) with each other. In physical terms, this unit can be represented with a macroscopic wave function Ψ (r, t).

This last mathematical step may seem somewhat audacious in form, but in essence it represents a coherent unit, where the individual parts are all in *phase*; that is, represented by a wave function where the elements that constitute it are all in the same “state.” In practice, we are dealing with macroscopic and coherent quantum waves of matter, to which the laws of optics ultimately also apply.

In quantum mechanics, a set of N identical particles where N is a sufficiently large (such as an ensemble of transient Sodium ions) is called a *many-body system*. It is characterized by a wave function that depends on the coordinates of the N particles and on time, i.e., Ψ = Ψ (r_1_, r_2_, …, r_N_, t): where for simplicity we have omitted the spin of the particles.

If the N particles are identical bosons, then the wave function written above is symmetric when two coordinates are swapped, i.e., Ψ (r_1_, r_2_, r_3_, …, r_N_, t) = Ψ (r_1_, r_2_, r_3_, …, r_N_, t). If, on the other hand, the N particles/atoms are identical fermions, then the wave function is antisymmetric when two coordinates are swapped, i.e., Ψ (r_1_, r_2_, r_3_, …, r_N_, t) = −Ψ (r_1_, r_2_, r_3_, …, r_N_, t). The resulting many-body wave function (returning to the transient ions) meets the requirements of a Schrödinger equation of the type: *i ^–^h* $\frac{\partial }{\partial t}$ Ψ(r_1_, r_2_, …, r_N_, t) = Ĥ Ψ (r_1_, r_2_, …, r_N_, t). Where Ĥ is the Hamiltonian operator of the t system.

This brief foray into quantum formalism provides us with an opportunity to discuss the well-known GRW theory of quantum mechanics in the context of the arguments we intend to put forward. This is not, of course, the place to explore this interpretation in depth. For our purposes, it is sufficient to know that it resolves a number of quantum problems related to the linearity of the Schrödinger equation, the collapse of the wave function, and the definition of the dynamics that characterize the “state” of microscopic and macroscopic systems. It should be noted that, from a predictive point of view, the GRW interpretation is equivalent to standard quantum theory, so it does not require any particular “logical leaps” to be accepted and/or understood. In his article “Are there quantum jumps?” (Bell, 1987), John Bell, one of the great theoretical quantum physicists (who supported the theory from the outset), recognized it as “a very nice illustration of how quantum mechanics, to become rational, requires only a change which is very small.”

But Why is the GRW theory ultimately so important for the arguments we intend to put forward in this article?

Firstly, because for the GRW model, the temperature value at which quantum systems are found (−270 °C or 37 °C) is not a fundamental parameter. Even the state of superposition/coherence in quantum ensembles in the phase preceding the spontaneous (and indeterministic) collapse of their wave function is not conditioned by size: in fact, for a fraction of a second, even the macroscopic quantum systems considered are in a genuinely superposed/coherent state. Admittedly, it must be pointed out that the GRW theory holds that in the fraction of a second mentioned above, there is only room for a few fleeting phenomena. We will, however, demonstrate that for durations on the order of a hundredth of a second, the synchronization of a certain number of “biological systems” like neurons can give rise, for example, to an NCC.

On the subject of the time frame that defines superimposed/coherent quantum states in the GRW model, Bell writes in the same article cited a few lines above (see Bell, 1987; p. 204): “the cat (referring to Schrödinger’s famous cat paradox) is not both dead and alive for more than a split second”. In our view, this is sufficient time to see a considerable amount of “work” come to fruition.

The spontaneous collapse of the wave function that characterizes the GRW theory of quantum mechanics also resolves the domino effect linked to the linearity of the Schrödinger equation and, by avoiding the pervasive effects of decoherence, also eliminates the problems associated with the interaction of quantum systems with the external environment. In more technical terms, the tendency towards spontaneous localisation of the elementary units of quantum systems (in particular transient sodium ions in a superimposed/coherent) is independent of any interaction with environmental conditions.

So, returning to the quantum dynamics that characterize the *synchronization* of synapse activation and Sodium ion flows that enter the depolarisation phase in neurons through the postsynapses, any uncertainty finds a rational explanation with the GRW model.

The hypothesis of spontaneous wave function collapse introduced by GRW, which depends on the number (N) of elementary units, suggests that a mental process – and, once again, we are extending the GRW model to neuroscience – can only manifest itself when a certain number of ions in a superimposed/coherent state (ions that, as we saw in Section 1, determine, after a series of formal steps, the number of neurons in a specific brain area responsible for the NCC) reaches a “critical” threshold. The value of 45,800 neurons may represent the threshold value at or above which the *spontaneous collapse* of wave function in a given cortical area is triggered, and thus a mental process is activated.

The minimum number (N) of elements that, according to GRW, is responsible for the spontaneous collapse of the wave function of a given system must be approximately on the order of Avogadro’s number. The mathematical formulation of the GRW model, which makes all this explicit, predicts that the wave function Ψ = Ψ (r_1_, r_2_, …, r_N_, t) of the system under consideration (see the formulation of the *many-body system* described above) will evolve in the usual way according to the Schrödinger equation. With a number of elements N on the order of Avogadro’s number, however, there is a very high probability that a discontinuous, spontaneous and acausal transition of the system itself may occur. The probability per unit of time of such a transition depends on the number of particles or atoms involved in the process, their spatial location and time.

The number N in the GRW model may correspond to the value N√N from Section 1, i.e., 9.62 × 10^13^, which does not have the magnitude of Avogadro’s number but still represents a considerable number of “elements.” The choice of value N√N as the threshold value that determines the spontaneous collapse of the wave function of a superimposed/coherent state of transient ions in a given area seems almost logical. On closer inspection, the “coefficient N√N” is the real key to determining the NCC. This coefficient represents both the diffusion parameter of the excited/coherent state in a given neural network and the overall “critical” number of transient Sodium ions involved in an NCC.

The formulation proposed by the GRW model and the coefficient N√N will also help us define the duration of an NCC. To do this, it is necessary to introduce a new constant of nature, as suggested in the original GRW article. This is the value λ (equal to 10^–16^), which represents the probability of a single particle (or atom) undergoing localisation (i.e., spontaneous collapse) in 1 s.

The process to which the overall wave function is subjected implies that the average lifetime, before a discontinuity, i.e., before a spontaneous collapse, will be given by the number of elementary units N of the system considered, multiplied by λ. In this case, the constant λ is *interpreted*, for all practical purposes, as one of the terms of a “cumulative time.” Essentially, λ becomes a characteristic time interval, Δ*t*, associated with a Sodium ion. Therefore, if we want to extend the formulation proposed by GRW to the field of neurophysiology and calculate the average duration of an NCC starting from the value “N” (equal to N∫N) of elements in a coherent quantum state and corresponding to 9.62 × 10^13^ (see Section 1), it will be sufficient to multiply this latter value by λ. The average time per NCC will be approximately equal to 0.01 s.

This value seems plausible to us, taking into account the electrical and chemical processes in the synapses and neurons of a specific cortical area. Similarly, the values assigned to the number of synapses, the number of (Na^+^) selectively “activable” ion channels per postsynaptic area and the flow of transient ions in the depolarisation phase defined in Section 1, appear to be well-calibrated, given that these values, after successive calculations of N_tot_ (referring to a neuron) and N∫N (referring to a “coherent” network of neurons), give a result of 9.62 × 10^13^, which multiplied by λ gives, as we have just seen, an acceptable value (one hundredth of a second) for the NCC.

To summarize, we can assert that when −0.01 s after the initiation of a quantum coherent process – approximately 45,800 neurons (this value, as we have seen, derives from a series of formal steps, each with a specific meaning) reach an extended coherent state, there is a *spontaneous reduction* in the system’s wave function and the mental process (NCC) takes shape.

We are now approaching the conclusion of this complex Section 2. The final topics we will cover concern what is known in neuroscience as the “binding problem” and the nature of consciousness.

The *binding problem* concerns how the brain unifies different perceptions into a single coherent experience, and more generally, how its vast neural networks communicate and coordinate. The visual system, for example, has specific areas in the brain responsible for interpreting various aspects of objects such as color, shape, movement, lines, angles, etc. When we see an object, we have a unified experience. How does the brain connect all these different stimuli into a unified conscious experience of the object? In his book The Astonishing Hypothesis: The Scientific Search for the Soul, (Crick, 1995), Francis Crick writes that the binding problem is: “the problem of how these neurons temporarily become active as a unit.”

Singer (1993), Singer and Gray (1995), at the University of Frankfurt maintain that the solution to the binding problem may lie in the synchronization of spatially separated neurons that are responsible for the various characteristics of an object [a view shared by Crick (1995)]. The neurons responsible for the characteristics of shape, color, movement, etc., are said to be excited in synchrony at a frequency of approximately 40 stimulations per second (40 Hz).

The prominent biologist and geneticist at the University of Surrey, McFadden (2013a,b), identifies the electromagnetic field generated by the brain’s electrical activity (frequency waves from 1 to 200 Hz detectable in EEGs) as responsible for synchronizing the widespread activation of cortical neurons, thereby resolving the binding problem in one fell swoop, as well as accounting for the transition process from unconscious to conscious thought. Similar ideas are also put forward by Fröhlich and McCormick (2010) in a much-debated article.

However, neither Singer, McFadden, Frohlich nor McCormick adequately address the extremely low energy of the brain’s electromagnetic waves –oscillating between 40 and 200 Hz – which they reference in their publications.

The individual particles (photons) of a 100 Hz electromagnetic wave, for example, generated by the brain’s electrical activity, have an energy, according to the well-known formula E = *h f*, of 6.62 × 10^–32^ J (i.e. 0.0000000000000000000000000000000662 J) and cannot, of course, even if there are a multitude of particles involved, influence the electrochemical activity of the cerebral cortex as a whole. Furthermore, these electromagnetic waves are not in phase, they are not coherent, and therefore, unlike photons emitted by lasers (made up of coherent quantum waves with remarkably high energy per unit of time), they cannot affect deeper brain processes. Electromagnetic waves (Gamma, Beta, etc.), ultimately, can only be a reflection of electrical activity in the brain and never the engine that synchronizes it, much less the cause of cognitive activity.

A possible solution to the binding problem and the nature of consciousness could come from the hypotheses we have put forward regarding the effects of quantum coherence extended to a single neuron and a network of neurons in the cortex. It has been repeatedly emphasized in this work that quantum coherence (with oscillation frequencies around 50 billion Hz, compared to 100 Hz for “classical” electromagnetic brain waves) can overcome synaptic barriers between neurons and extend not only to specific cortical areas, but also to the entire brain.

Before addressing these dynamics in detail, it is important to point out that, in our view, none of the hypotheses put forward by a significant number of neuroscientists, biologists and physicists regarding non-local quantum action to explain the binding problem (and the neural correlate of consciousness) can be deemed acceptable. Systems such as Sodium ions in the depolarisation phase can easily exploit (in addition to coherence) quantum non-locality (see Bernroider and Roy, 2005; Bernroider and Summhammer, 2012; Salari et al., 2015; Summhammer et al., 2018) for the simple reason that they all essentially “originate/belong” to the same neuron: that is, they have their origin in a specific “place.” Only by hypothesizing a shared origin is it possible to imagine a dynamic in which individual elementary units evolve non-locally (or entangled). In this regard, it is worth considering the experiments conducted since 1982 by Aspect et al. (1982) and others, which have confirmed quantum entanglement (Stella et al., 2022). In these tests, the French scientist verified entanglement starting from a pair of correlated particles generated by a single calcium atom.

To explain the binding problem, in our view, it is necessary to bring into play a mechanism that “arises” and extends *simultaneously* in different (separate) cortical areas. Such a mechanism involving areas without a precise initial link can only arise from extended *quantum coherence*.

In this sense, all the scientists mentioned above [from Crick (1995) to McCormick] were right to turn to a *mechanism* acting throughout the brain to explain the binding problem or the neural correlate of consciousness. Unfortunately, the “classical” brain waves they invoke not only lack the required speed of action and are not “coherent,” but also have such low energy that they cannot possibly influence the electrochemical activity of the brain.

Quantum coherence, on the other hand, is expressed through very high oscillation frequencies: 50 GHz (in a simulation, the Salzburg and Vienna group, as previously noted, went so far as to estimate oscillation frequencies of transient ions of 900 GHz – and frequency is directly proportional to energy). This energy can undergo a further positive variation in quantum coherent systems as a result of the wave phase concordance of the systems themselves; however, so as not to overly complicate this discussion, we will not address this important argument here.

Let us now return to the eyes of someone looking at an object that has shape, color, movement, etc., and which, through a complex system (starting from the photoreceptors in the retina and continuing in the optic nerve), “activate” – almost simultaneously – the neurons in certain areas of the cerebral cortex through a process of extended quantum coherence. Is it possible, following our line of research, to quantify the time and number of neurons involved in this unified visual experience?

The answer is yes. The formal steps (partly derived from Section 1) for defining the time and minimum number of neurons in different brain areas involved in a standard visual experience are as follows. In the first step, we need to multiply the number 9.62 × 10^13^ (i.e., the N√N of a standard NCC) by the number of areas involved in a single coherent experience such as seeing an object: let us assume 10. The result will be the *total* number of coherent transient Sodium ions involved in the process of seeing an object: 9.62 × 10^14^. Multiplying this value by the constant λ introduced by GRW, i.e., 10^–16^, gives us the average time for the mental process involved in seeing an object: approximately 0.096 s. Finally, by repeating the formal steps of Section 1, we can also calculate the *minimum* number of neurons involved in the mental process of sight: i.e., 458,161. It is worth pointing out that this value concerns the perception/processing of a “simple” image. For the processing of *complex images*, we estimate that this value could increase by a factor of 1,000 or perhaps even 10,000. Thus, for a mental process focused on seeing a “complex” object, the number of neurons involved, throughout the 10 (or more) areas of the cortex previously considered, could increase to 458,161,000, up to a maximum of 4,581,610,000. Unfortunately, with these numbers involved, it is impossible (following the formulations we have suggested thus far) to calculate the *average time* taken for a complex visual process to occur, i.e., the duration of the synchronized coherent state that subsequently resolves itself with spontaneous collapse, and therefore with the realization of a visual process.

The quantum coherence that synchronizes the “discharges” in the 10 areas considered for the visual processing of a “simple” image and that allows this image to be placed in time and space suggests something more than just cold numbers. Indeed, the dynamics described above could represent the neural correlates of *visual consciousness*.

Consciousness can manifest itself in myriad ways. Moreover, since the total sum of neurons that are activated (or are not activated, bearing in mind that the quantum mind model is “indeterministic” and not computational) must somehow encode our thoughts, then these same thoughts can be reflected and encoded in the sum of all the quantum ion flows that enter the depolarisation phase from the postsynaptic membranes, and then dynamically integrate with the systems present within the neuron.

In conclusion, due to its wave-particle characteristics, extended quantum coherence would synchronize (making them coherent) all the ions and all the ion channels of different parts of the brain, playing a decisive role in the transition between unconscious and conscious thought, and between our unconscious and conscious behaviors. We can venture the hypothesis (though it is pure speculation) that the brain is a macroscopic quantum and “classical” super system: functioning as a filtering structure (macroscopic) that selects background probabilistic oscillations (microscopic), “accepting” those experienced as useful and positive and rejecting those that are negative. This fact would also explain the evolutionary plasticity of the human personality.

## Section 3. Identification of the characteristic resonance frequencies of neurons and therapeutic implications

Based on the arguments presented thus far, it can be hypothesized that there is a real possibility that cellular activity as a whole is underpinned by a process of *biological quantum coherence* that manifests itself through *resonance oscillations* in a range between 10^10^ and 10^11^ Hz.

In this regard, a series of experiments could be carried out involving the irradiation of live cell cultures *in vitro* (cortical neurons), using generators that produce high-frequency waves (between 42 and 69 GHz), with very low power levels (<10 mW/cm^2^) so as to avoid thermal effects – in order to highlight, through morphological, metabolic and genetic mutations in the irradiated cellular material, the typical resonance frequencies for each cell. Subsequently, building on and *interpreting* the collected data, tests could be performed by irradiating cultures of diseased neurons, obtained, for example, from human induced pluripotent stem cell (iPSC) cultures. Stem cell cultures, when appropriately processed, are capable of reproducing pathologies such as Parkinson’s, Alzheimer’s, and ALS *in vitro* (Limone et al., 2022; Cerneckis et al., 2024) and irradiation with specific oscillation frequencies (derived by integrating the frequency values identified in the first phase of experimentation with mathematical models typically used in neurobiology and cytology) could produce positive changes in the cell sample studied. The final step could be to apply the knowledge acquired to the treatment of people affected by certain neurodegenerative diseases.

A paradigmatic example of the modus operandi that should characterize the various experimental phases comes from the work of two research groups at the Institute of Physics and the Institute of Genetics at the University of Stuttgart, composed of physicists F. Kremer, L. Santo, A. Poglitsch and geneticists C. Koschnitzke and P. Quick. Their research, conducted in 1981, had the ultimate goal of highlighting possible “mutations” in a biological sample composed of giant chromosomes taken from the salivary glands of *Acricotopus lucidus* midges. This sample was subjected to irradiation with electromagnetic waves in the range of 64–69 GHz, but with an extremely low power density <5 mW/cm^2^ (for a schematic description of the equipment used, see Figure 1). The report of these experiments was presented at the International Symposium held in Bad Neuenahr (Fröhlich and Kremer, 1983; pp. 10–20) mentioned in Section 1. Although these works might be seen as somewhat dated, the methodologies and scientific rigor employed still serve as a point of reference for any research in this field. In dedicated two-hour-long lab sessions, the researchers at the University of Stuttgart found clear “mutations” in the irradiated biological material sample. However, these “mutations” only occurred at specific radiation frequencies and, given the very low power density levels used, and therefore with negligible thermal effects, they concluded that the observed “mutations” must be based on a process of *biological quantum coherence* expressed by *resonance oscillations* in a range between 64 and 69 GHz.

**FIGURE 1:**
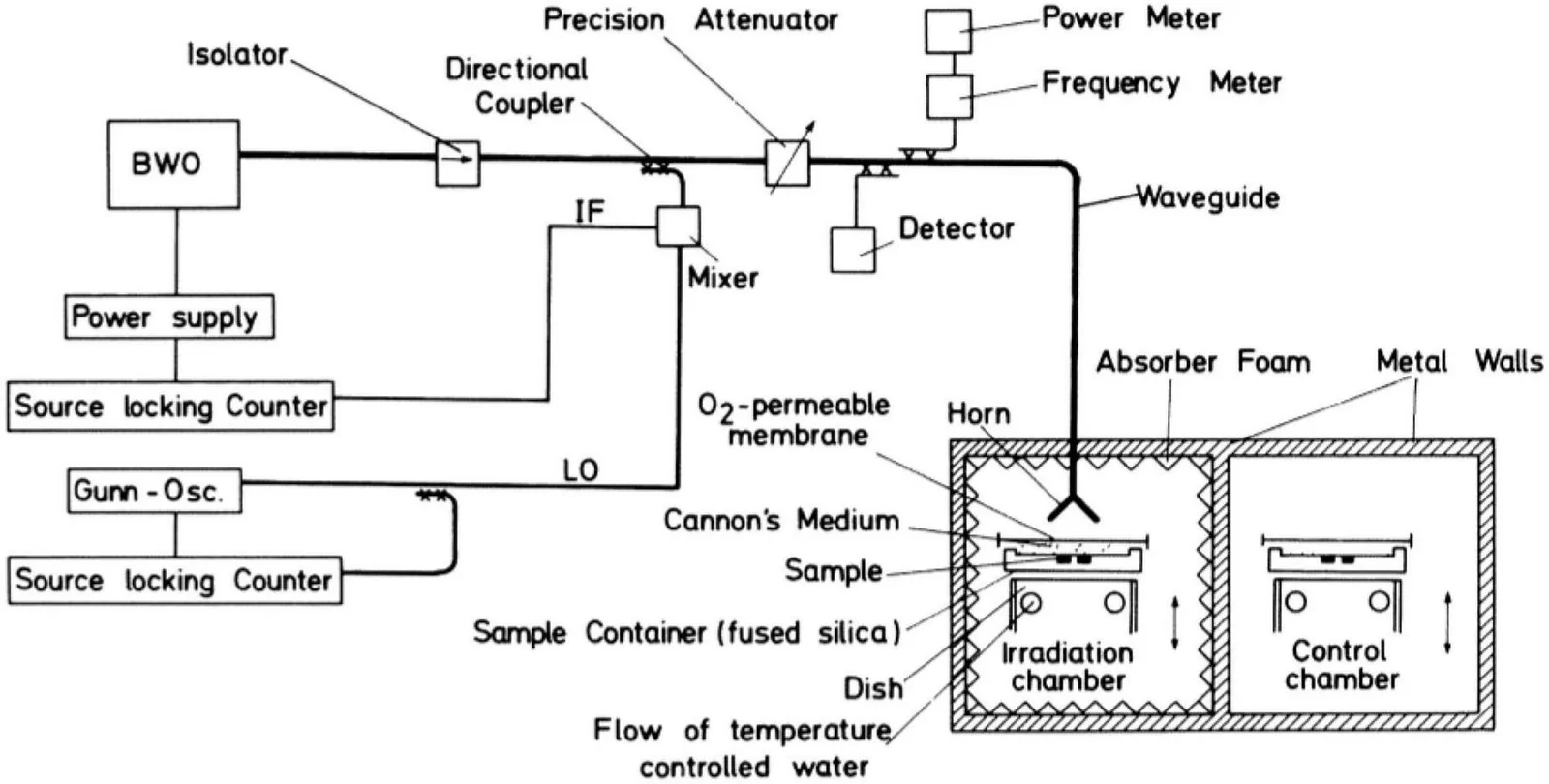
Diagram of the equipment used by researchers at the University of Stuttgart to highlight the “mutations” in the biological samples under study.

If it were to be experimentally confirmed that individual neurons or neural networks can exhibit phenomena of extended quantum coherence (and, once again, we return to the field of neurobiology), this could open up new perspectives in neuroscience (and other fields) for understanding and treating a wide range of pathologies. The study group led by the well-known neurophysiologist Llinas et al. (2002) proposed a particular method of treating specific brain pathologies with high-frequency electromagnetic waves. In one of his articles (Llinas et al., 2002), the American scientist suggests that stimulating the deep regions of the brain with waves of a certain frequency can produce clear therapeutic effects on stereotaxic brain lesions.

To date, however, no serious attempts have been made to identify the exact resonance frequencies typical of healthy neurons (frequencies that can be used secondarily to irradiate diseased neurons and return them to their natural oscillation frequency). Sporadic attempts at treatment have been conducted by exposing patients to electromagnetic fields – especially in Transcranial Magnetic Stimulation (Koch et al., 2025) – at low frequencies. However, high frequencies such as those proposed in the current study, i.e., between 42 and 69 GHz, have never been used. Only the identification of the exact resonance frequencies within this range could potentially produce beneficial effects on the neural networks involved in certain degenerative pathologies such as Alzheimer’s, Parkinson’s, etc. A better understanding of the processes underlying neuronal activity, moreover, could lead to a deeper understanding of cognitive brain dynamics as a whole.

A final consideration, which however, goes beyond the primary objectives of this work, concerns the possibility of verifying, with the experiments proposed thus far, the effects of electromagnetic waves from latest generation mobile phones (which can currently reach oscillation frequencies of 52 GHz), on the samples of cellular material being studied.

## Data Availability

The raw data supporting the conclusions of this article will be made available by the author, without undue reservation.
